# Evaluation of entropy driven jet symmetry transitions

**DOI:** 10.1038/s41598-022-13161-w

**Published:** 2022-08-02

**Authors:** Leonard F. Pease, Lenna A. Mahoney, S. Thomas Yokuda, Judith A. Bamberger, Michael J. Minette

**Affiliations:** Pacific Northwest National Laboratory (PNNL), 902 Battelle Boulevard, MSIN K9-89, P.O. Box 999, Richland, WA 99352 USA

**Keywords:** Fluid dynamics, Mechanical engineering

## Abstract

Here we determine whether entropy drives planar turbulent jets into round turbulent jets. Determining when a jet flow transitions from one symmetry to the next is an important but incompletely resolved problem. The constructal view argues that the transition between symmetries of jet flows is governed by the minimization or maximization of entropy. Here we explore whether entropy increases with the transition of a planar turbulent jet into a round turbulent jet and whether entropy maximization (or minimization) predicts the same location of symmetry transition as velocity matching. We find that entropy considerations presented do not predict this transition.

## Introduction

Why turbulent jets change symmetry is an open question. For example, rectangular turbulent jets become turbulent circular jets a suitable distance away from the orifice. That transition is particularly interesting because it is not induced by interaction with a surface (e.g., circular turbulent jets transforming into radial wall jets). However, why this transition occurs in the absence of a surface remains unclear.

Bejan et al.^1^ argue that observation of turbulent planar jets morphing into round turbulent jets is an example of evolution. They support this argument by contending that this change in jet symmetry increases jet mixing as an example of “nature’s arrow”, phenomena traditionally associated with entropy. Yet, Bejan et al., are careful to not use the term entropy, arguing from a constructal view that entropy must only remain positive for irreversible processes, and use velocity matching alone to determine the location of the symmetry transition without reference to entropy development. Heitor Reis argues that minimization or maximization of entropy are direct corollaries of the constructal view, suggesting that entropy (or a rate thereof) may play an essential role in the transition between jet symmetries^2^.

Inspired by this potential explanation of jet symmetry transition, here we explore whether entropy increases with the transition of a planar turbulent jet into a round turbulent jet and whether entropy maximization (or minimization) predicts the same location of symmetry transition as velocity matching. We find that entropy considerations presented do not predict this transition.

## Model

Consider, as do Bejan et al.^1^ fully immersed turbulent jets in single component fluids (e.g., water) of infinite domain. The jet comprises the system of interest. Entropy is related to internal energy, pressure, and volume through

1 $$TdS = dU + pdV,$$

where *T* is the absolute temperature, *S* is the specific entropy, *U* is the specific internal energy, *p* is pressure, and *V* is the specific volume (inversely proportional to the density *ρ*). Application of the total derivative (*D*/*Dt*) indicates

2 $$\rho T\frac{DS}{{Dt}} = \rho \frac{DU}{{Dt}} - \frac{p}{\rho }\frac{D\rho }{{Dt}}$$

within the control volume defined by the jet, where *t* is time. Internal energy conservation may be expressed as

3 $$\rho \frac{DU}{{Dt}} = - \nabla \cdot {\mathbf{\varvec{q}}} - p\nabla \cdot {\mathbf{\varvec{v}}} + {{\varvec{\uptau}}}:\nabla {\mathbf{\varvec{v}}},$$

where ***q*** is the heat flux, ***τ*** is the deviatoric stress tensor, and ***v*** is the local fluid velocity vector^3^. Substitution and simplification for isothermal jets in the absence of mass sources or sinks (e.g., suction)^4^ then requires

4 $$\frac{DS}{{Dt}} = \frac{{{{\varvec{\uptau}}}:\nabla {\mathbf{\varvec{v}}}}}{\rho T},$$

showing that this double dot product is the source of entropy. The double dot product must be positive as minimally required by the second law of thermodynamics. Yet, if the argument that entropy drives the jet transition holds, then a transition that maximizes (or minimizes) the double dot product would be preferred. The remainder of this article evaluates this proposition.

For an incompressible isothermal fluid, where the divergence of the velocity vanishes due to mass conservation,

5 $${{\varvec{\uptau}}}:\nabla {\mathbf{\varvec{v}}} = \mu \left( {2\Gamma } \right)^{2}$$

with *μ* here specified as the local turbulent viscosity (i.e., for moderate to low viscosity fluids) and *Γ* is the magnitude of the rate of strain tensor^3^. The square indicates that viscous dissipation is always positive as expected. We now evaluate the double dot product for the planar and round free turbulent jets.

Consider then a planar turbulent jet issuing in the stream wise *z* direction from a slot of length *L* and width *W*, where *L* ≫ *W*, with spreading in the *x* direction in Cartesian coordinates. With velocity and derivatives in the *y* direction (perpendicular to *z*) vanishing (i.e., *v*_*y*_ = *d*/*dy* = 0),

6 $${{\varvec{\uptau}}}:\nabla {\mathbf{\varvec{v}}} = 4\mu \left( {\frac{{\partial v_{z} }}{\partial z}} \right)^{2} + \mu \left( {\frac{{\partial v_{z} }}{\partial x} + \frac{{\partial v_{x} }}{\partial z}} \right)^{2},$$

simplified at constant density with continuity as

7 $$0 = \frac{{\partial v_{z} }}{\partial z} + \frac{{\partial v_{x} }}{\partial x} ,$$

where the subscript indicates a component in the direction indicated. The error in this expression relative to the three-dimensional version is *O*((*W*/*L*)^2^) where *O* is the order operator, so that when *L* ≫ *W* the error introduced by the simplification is quantitatively small. Because the velocity is turbulent, it may be divided into both time-smoothed averages and fluctuations relative to the average with *v*_*i*_ =  < *v*_*i*_ >  + *u*_*i*_, with < *u*_*i*_ >  = 0, where *i* = {*x*, *z*} and < … > indicates temporal averaging. Applying the averaging operator as defined by Deen^3^ returns

8 $$\left\langle {{{\varvec{\uptau}}}:\nabla {\mathbf{\varvec{v}}}} \right\rangle = 4\mu \left( {\frac{{\partial \left\langle {v_{z} } \right\rangle }}{\partial z}} \right)^{2} + \mu \left( {\frac{{\partial \left\langle {v_{z} } \right\rangle }}{\partial x} + \frac{{\partial \left\langle {v_{x} } \right\rangle }}{\partial z}} \right)^{2} + 4\mu \left\langle {\left( {\frac{{\partial u_{z} }}{\partial z}} \right)^{2} } \right\rangle + \mu \left\langle {\left( {\frac{{\partial u_{z} }}{\partial x} + \frac{{\partial u_{x} }}{\partial z}} \right)^{2} } \right\rangle ,$$

which shows that in addition to two terms for time-averaged velocities, two additional terms with instantaneous velocities also contribute. The time-average planar jet velocity profile has long been known to be self-similar sufficiently far away from the issuing orifice, with the velocity in the *z* direction as

9 $$\left\langle {v_{z} } \right\rangle = \frac{{h_{p} U_{o} W^{1/2} }}{{z^{1/2} }}f_{p} = v_{{z{\text{,max}}}} f_{p} ,$$

where the second equality defines *v*_*z,max*_ for a planar jet, *h*_*p*_ is the velocity-decay constant for the planar jet, *U*_*o*_ is the average nozzle velocity, *W* is the nozzle width (not half width), and *f*_*p*_ is the velocity distribution for the planar jet as a function of both *x* and z^5^. The impact of uncertainty from the self-similarity assumption is evaluated a posteriori. To first order approximation, *f*_*p*_ is a Gaussian distribution that depends on a similarity parameter *η*_*p*_ = *x*/*β*_*p*_*z*, where *β*_*p*_ represents the jet spread of the planar jet. Mass conservation through the continuity equation specifies

10 $$\left\langle {v_{x} } \right\rangle = \frac{{h_{p} U_{o} W^{1/2} }}{{z^{1/2} }}\beta_{p} \int\limits_{0}^{{\eta_{p} }} {\left( {\frac{{f_{p} }}{2} + \eta_{p} \frac{{df_{p} }}{{d\eta_{p} }}} \right)d\eta_{p} }$$

with use of symmetry to set the lower limit of integration. Rajaratnam^5^ gives the turbulent viscosity using the Prandtl-Boussinesq eddy viscosity formulation as

11 $$\mu = \rho k_{p} h_{p} U_{o} \beta_{p} \sqrt {Wz},$$

where *k*_*p*_ is a constant. The fluctuating velocities may be approximated to first order as *u*_*i*_ ~ (*u*_*i*_^2^)^1/2^. Experimental data reported by Heskestad^6^ and Rajaratnam^5^ suggests *u*_*z*_ ~ (*u*_*z*_^2^)^1/2^ = *f*_*z*_^*p*^*v*_*z,max*_ and *u*_*x*_ ~ (*u*_*x*_^2^)^1/2^ = *f*_*x*_^*p*^*v*_*z,max*_. Expressions for *f*_*z*_^*p*^ and *f*_*x*_^*p*^ are fit in Fig. 1a. Substitution and integration over the traverse directions then returns

12 $$\left\langle {2\int\limits_{0}^{L} {\int\limits_{0}^{\infty } {{{\varvec{\uptau}}}:\nabla {\mathbf{\varvec{v}}}dxdy} } } \right\rangle = 2\frac{{\rho k_{p} h_{p}^{3} U_{o}^{3} \beta_{p}^{2} W^{3/2} L}}{{z^{3/2} }}\left( {I_{p}^{a} + I_{p}^{f} } \right),$$

which shows that viscous dissipation and entropy generation (at constant *ρT*) decay as *z*^− 3/2^ for the planar turbulent jet. The constants *I*_*p*_^*a*^ and *I*_*p*_^*f*^ evaluate to

13 $$I_{p}^{a} = \int\limits_{0}^{\infty } {\left[ {4\left( {\frac{{f_{p} }}{2} + \eta_{p} \frac{{df_{p} }}{{d\eta_{p} }}} \right)^{2} + \left( {\frac{1}{{\beta_{p} }}\frac{{df_{p} }}{{d\eta_{p} }} - \beta_{p} \eta_{p} \left( {\frac{{f_{p} }}{2} + \eta_{p} \frac{{df_{p} }}{{d\eta_{p} }}} \right) - \frac{{\beta_{p} }}{2}\int\limits_{0}^{{\eta_{p} }} {\left( {\frac{{f_{p} }}{2} + \eta_{p} \frac{{df_{p} }}{{d\eta_{p} }}} \right)} d\eta_{p} } \right)^{2} } \right]} d\eta_{p} .$$

and

14 $$I_{p}^{f} = \int\limits_{0}^{\infty } {4\left( {2\eta_{p}^{2} g_{z}^{p} - \frac{{f_{z}^{p} }}{2}} \right)^{2} + \left( {2\eta_{p}^{2} g_{x}^{p} - \frac{{f_{x}^{p} }}{2} - \frac{{2\eta_{p} }}{{\beta_{p} }}g_{z}^{p} } \right)^{2} d\eta_{p} } ,$$

where for convenience, we have defined

15 $$f_{i}^{p} = \sum\limits_{j = 1}^{2} {\left( { - 1} \right)^{j + 1} A_{ij}^{p} Exp\left[ { - B_{ij}^{p} \eta_{p}^{2} } \right]}$$

and

16 $$g_{i}^{p} = \sum\limits_{j = 1}^{2} {\left( { - 1} \right)^{j + 1} A_{ij}^{p} B_{ij}^{p} Exp\left[ { - B_{ij}^{p} \eta_{p}^{2} } \right]} ,$$

where the index *i* takes on *x* and *z*, and index *j* is numerical. For a Gaussian distribution of *f*_*p*_ = Exp[− Ln(2)*η*_*p*_^2^],

17 $$I_{p}^{a} = \frac{1}{2}\sqrt {\frac{\pi }{{{\text{Ln}}\left( 4 \right)}}} + \frac{1}{{\beta_{p}^{2} }}\sqrt {\frac{{\pi {\text{Ln}}\left( 2 \right)}}{8}} - 1.016\beta_{p}^{2}$$

over *η*_*p*_ = [0, ∞) for the first two symbolic terms and, equivalently, *η*_*p*_ = [0, 3) for the final numerical term (because the Gaussian distribution representing the velocity profile vanishes beyond *η*_*p*_ = 3 so must expressions that depend directly on the gradient in velocity). For a planar free turbulent jet^5^, *h*_*p*_ = 2.21–2.67 based on full nozzle width, *β*_*p*_ = 0.097–0.114, and *k*_*p*_ = 0.0373–0.0438 giving *I*_*p*_^*a*^ = 40.9–56.2. For the distributions in Fig. 1a, *I*_*p*_^*f*^ = 3.65, which remains nearly an order of magnitude smaller, suggesting that instantaneous terms remain secondary quantitatively.

In contrast, consider a round turbulent jet issuing in the *z* direction from a nozzle of diameter *d*_*o*_ with *r* radially transverse to the jet’s axis of symmetry in cylindrical coordinates with velocity and derivatives in the azimuthal direction vanishing (i.e., *v*_*θ*_ = 0, *d*/*d*θ = 0, (∇***v***)_*θθ*_  = 0),

18 $${{\varvec{\uptau}}}:\nabla {\mathbf{\varvec{v}}} = 2\mu \left( {\frac{{\partial v_{r} }}{\partial r}} \right)^{2} + 2\mu \left( {\frac{{\partial v_{z} }}{\partial z}} \right)^{2} + \mu \left( {\frac{{\partial v_{z} }}{\partial r} + \frac{{\partial v_{r} }}{\partial z}} \right)^{2}.$$

B﻿ecause the velocity is turbulent it may be divided into both time-smoothed averages and fluctuations relative to the average with *v*_*i*_ =  < *v*_*i*_ >  + *u*_*i*_, with < *u*_*i*_ >  = 0, where *i* = {*r*, *z*} and < … > indicates temporal averaging^3^. Applying the averaging operator as defined by Deen^3^ returns

19 $$\begin{gathered} \left\langle {{{\varvec{\uptau}}}:\nabla {\mathbf{\varvec{v}}}} \right\rangle = 2\mu \left( {\frac{{\partial \left\langle {v_{r} } \right\rangle }}{\partial r}} \right)^{2} + 2\mu \left( {\frac{{\partial \left\langle {v_{z} } \right\rangle }}{\partial z}} \right)^{2} + \mu \left( {\frac{{\partial \left\langle {v_{z} } \right\rangle }}{\partial r} + \frac{{\partial \left\langle {v_{r} } \right\rangle }}{\partial z}} \right)^{2} \hfill \\ + 2\mu \left\langle {\left( {\frac{{\partial u_{r} }}{\partial r}} \right)^{2} } \right\rangle + 2\mu \left\langle {\left( {\frac{{\partial u_{z} }}{\partial z}} \right)^{2} } \right\rangle + \mu \left\langle {\left( {\frac{{\partial u_{z} }}{\partial r} + \frac{{\partial u_{r} }}{\partial z}} \right)^{2} } \right\rangle , \hfill \\ \end{gathered}$$

which shows that both time-averaged velocities and instantaneous velocities contribute. The time-average round jet velocity profile has long been known to be self-similar sufficiently far away from the nozzle as

20 $$\left\langle {v_{z} } \right\rangle = \frac{{h_{r} U_{o} d_{o} }}{z}f_{r} = v_{{z{\text{,max}}}} f_{r},$$

where the second equality defines *v*_*z,max*_ for a round jet, *h*_*r*_ is the velocity-decay constant for the round jet, *U*_*o*_ is the average nozzle velocity, *d*_*o*_ is the nozzle width or diameter, and *f*_*r*_ is the velocity distribution of the round jet as a function of both *r* and *z*^5^. To first-order approximation, *f*_*r*_ is a Gaussian distribution as a function of the similarity parameter *η*_*r*_ = *r*/*β*_*r*_*z*, where *β*_*r*_ represents the jet spread for the round jet. Mass conservation through the continuity equation,

21 $$0 = \frac{{\partial rv_{z} }}{\partial z} + \frac{{\partial rv_{r} }}{\partial r},$$

specifies the radial velocity as

22 $$\left\langle {v_{r} } \right\rangle = \frac{{h_{r} U_{o} d_{o} }}{z}\frac{{\beta_{r} }}{{\eta_{r} }}\int\limits_{0}^{{\eta_{p} }} {\left( {f_{r} + \eta_{r} \frac{{df_{r} }}{{d\eta_{r} }}} \right)\eta_{r} d\eta_{r} }$$

with use of symmetry to set the lower limit of integration. Rajaratnam^5^ (see Eqs. 2-70 and 2-71) gives the turbulent viscosity using the Prandtl-Bousinesq eddy viscosity formulation as

23 $$\mu = \rho k_{r} h_{r} U_{o} d_{o} \beta_{r} ,$$

where *k*_*r*_ is a constant. The fluctuating velocities may be approximated to first order as *u*_*i*_ ~ (*u*_*i*_^2^)^1/2^. Experimental data reported by Wygnanski and Fiedler^7^ and Rajaratnam^5^ suggests *u*_*z*_ ~ (*u*_*z*_^2^)^1/2^ = *f*_*z*_^*c*^*v*_*z,max*_ and *u*_*r*_ ~ (*u*_*r*_^2^)^1/2^ = *f*_*r*_^*c*^*v*_*z,max*_. Expressions for *f*_*z*_^*c*^ and *f*_*r*_^*c*^ are fit in Fig. 1b.

**Figure 1:**
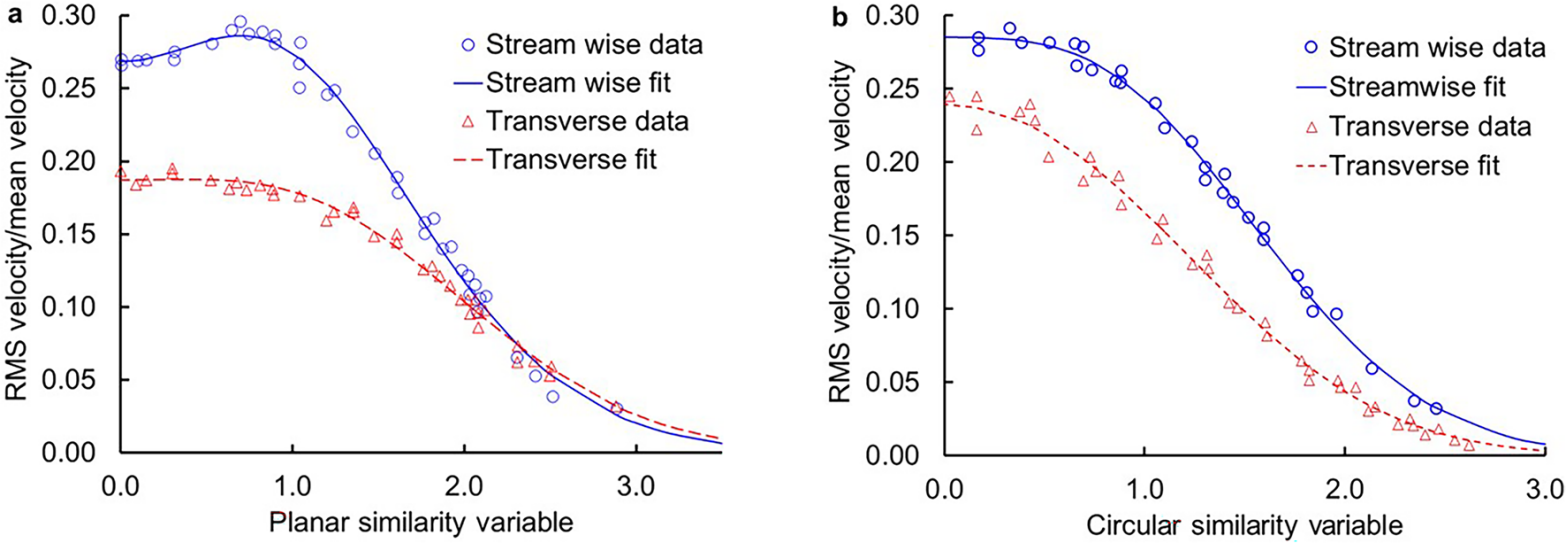
Fits of the time average (root mean square) fluctuation velocities scaled on the centerline mean velocity for (**a**) planar and (**b**) round jets. Fits in panel a include *f*_*z*_^*p*^ = 0.492Exp[− 0.353* η*_*p*_^2^] − 0.224Exp[− 1.14* η*_*p*_^2^] and *f*_*x*_^*p*^ = 0.837Exp[− 0.346* η*_*p*_^2^]− 0.650Exp[− 0.454* η*_*p*_^2^] and in panel b include *f*_*z*_^*c*^ = 0.815Exp[− 0.508* η*_*r*_^2^]− 0.529Exp[− 0.764*η*_*r*_^2^] and *f*_*r*_^*c*^ = 0.769Exp[− 0.566*η*_*r*_^2^]− 0.530Exp[− 0.671*η*_*r*_^2^] assuming *β*_*p*_ = 0.10 and *β*_*r*_ = 0.09. Data from Figs. 1-7c-d and 2-8b-c of Rajaratnam^5^.

Substitution and integration over the transverse direction leads to

24 $$\left\langle {2\pi \int\limits_{0}^{\infty } {{{\varvec{\uptau}}}:\nabla {\mathbf{\varvec{v}}}rdr} } \right\rangle = 2\pi \frac{{\rho k_{r} h_{r}^{3} U_{o}^{3} \beta_{r}^{3} d_{o}^{3} }}{{z^{2} }}\left( {I_{r}^{a} + I_{r}^{f} } \right) ,$$

which shows that viscous dissipation and entropy generation (at constant *ρT*) decay as *z*^−2^. The constant *I*_*r*_^*a*^ evaluates to

25 $$I_{r}^{a} = \int\limits_{0}^{\infty } {\left[ \begin{gathered} 2\left( {f_{r} + \eta_{r} \frac{{df_{r} }}{{d\eta_{r} }} - \frac{1}{{\eta_{r}^{2} }}\int\limits_{0}^{{\eta_{r} }} {\left( {f_{r} + \eta_{r} \frac{{df_{r} }}{{d\eta_{r} }}} \right)\eta_{r} d\eta_{r} } } \right)^{2} + 2\left( {f_{r} + \eta_{r} \frac{{df_{r} }}{{d\eta_{r} }}} \right)^{2} \hfill \\ + \left( {\frac{1}{{\beta_{r} }}\frac{{df_{r} }}{{d\eta_{r} }} - \beta_{r} \eta_{r} \left( {f_{r} + \eta_{r} \frac{{df_{r} }}{{d\eta_{r} }}} \right)} \right)^{2} \hfill \\ \end{gathered} \right]\eta_{r} d\eta_{r} } .$$

and

26 $$I_{r}^{f} = \int\limits_{0}^{\infty } {\left[ {2\left( {\frac{{2\eta_{r} }}{{\beta_{r} }}g_{r}^{c} } \right)^{2} + 2\left( {2\eta_{r}^{2} g_{z}^{c} - f_{z}^{c} } \right)^{2} + \left( {2\eta_{r}^{2} g_{r}^{c} - \frac{{2\eta_{r} }}{{\beta_{r} }}g_{z}^{c} - f_{r}^{c} } \right)^{2} } \right]\eta_{r} d\eta_{r} },$$

where for convenience, we have defined

27 $$f_{i}^{c} = \sum\limits_{j = 1}^{2} {\left( { - 1} \right)^{j + 1} A_{ij}^{c} Exp\left[ { - B_{ij}^{c} \eta_{r}^{2} } \right]}$$

and

28 $$g_{i}^{c} = \sum\limits_{j = 1}^{2} {\left( { - 1} \right)^{j + 1} A_{ij}^{c} B_{ij}^{c} Exp\left[ { - B_{ij}^{c} \eta_{r}^{2} } \right]},$$

where the index *i* takes on *r* and *z*, and index *j* is numerical. For a Gaussian distribution of *f*_*r*_ = Exp[− Ln(2)*η*_*r*_^2^], the constant reduces to

29 $$I_{r}^{a} = \frac{1}{{2\beta_{r}^{2} }} + \beta_{r}^{2} \frac{3}{{8{\text{Ln}}^{2} \left( 2 \right)}} + \frac{1}{2} .$$

over *η*_*r*_ ∈ [0, ∞). For a round free turbulent jet^5^, *h*_*r*_ = 5.75–7.32, *β*_*r*_ = 0.082–0.097, and *k*_*r*_ = 0.030–0.036 giving *I*_*r*_^*a*^ = 53.6–74.8. For the distributions in Fig. 1b, *I*_*r*_^*f*^ = 14.47.

## Results and discussion

The purpose of this article is to determine whether entropy governs the transition between hydrodynamic jet symmetries. The remainder of this article first determines whether the transition is related to maximization or minimization of entropy (mutually exclusive options) and second determines whether predicted transitions are in quantitative agreement with experiment.

**Figure 2:**
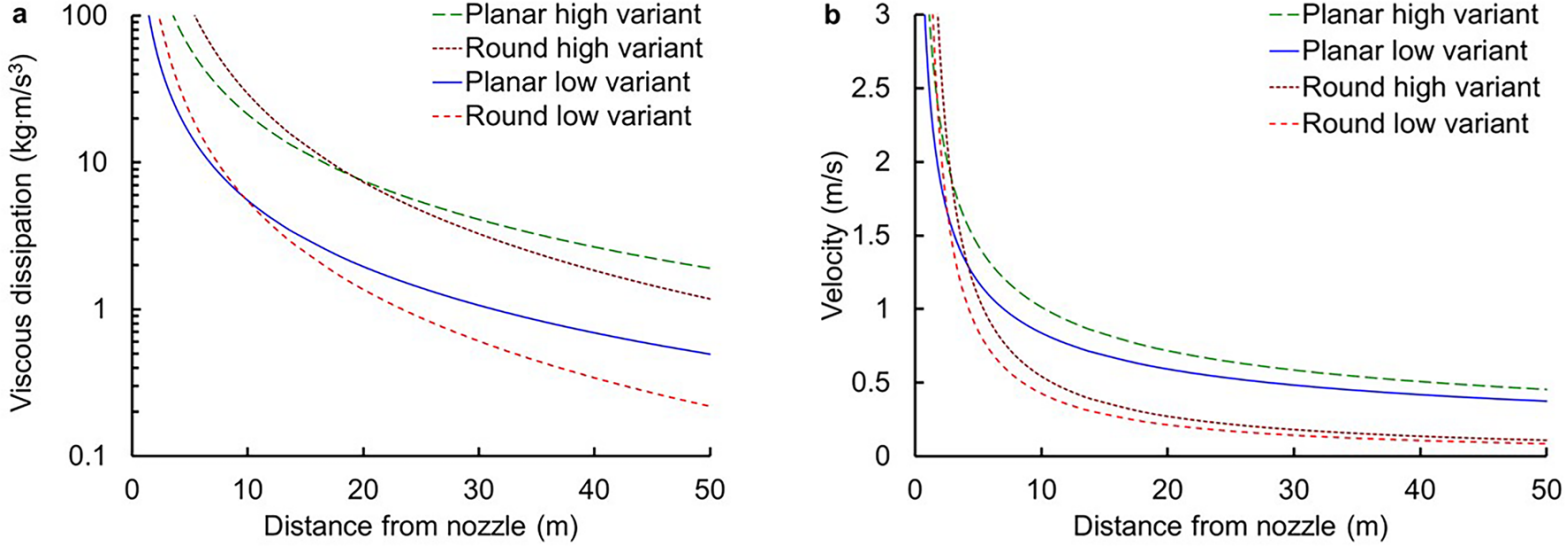
Viscous dissipation and centerline velocity versus distance from jet nozzle. (**a**) Viscous dissipation (equivalent to entropy generation at constant temperature and density) as a function of distance from the jet nozzle (from Eqs. 12 and 24) for *ρ* = 1000 kg/m^3^, *U*_*o*_ = 12 m/s, *W* = 0.01 m, *L* = 0.3 m, and *d*_*o*_ = (4*WL*/π)^1/2^ with other values as listed in the text. (**b**) Centerline velocity as a function of distance from the jet nozzle (from Eqs. 9 and 20) for the same values. The high and low variants use the parameter ranges in the text to bound the viscous dissipation and velocity.

Figure 2 presents viscous dissipation for both jets as a function of distance from the nozzle. Because viscous dissipation is positive for both jets, entropy is generated with both jets as anticipated from the second law of thermodynamics for irreversible processes. The figure shows that viscous dissipation decreases with distance from the nozzle. The round jet starts at a higher viscous dissipation rate but then decays more rapidly than the planar jet that starts at a lower viscous dissipation rate. If maximizing entropy generation (i.e., maximizing momentum mixing) were to have been the driver of the transition in jet symmetry then one would expect the viscous dissipation of the planar jet to exceed that of the round jet at short distances but the round jet to exceed the planar jet at large distances. However, the figure shows exactly the opposite. Therefore, the transition is not accompanied by an increase in entropy generation; entropy maximization does not govern the transition. This finding leaves the question of whether entropy minimization quantitatively predicts the transition as discussed below.

The difference in exponent on distance from the nozzle indicates an intersection of the curves. Forcing the two jets to have the same starting momentum π*ρU*_*o*_^2^*d*_*o*_^2^/4 = *ρU*_*o*_^2^*WL* in the limit of perfect plug flow at the nozzle finds entropy equivalence at

30 $$z_{transition} = \left[ {\frac{{64k_{r}^{2} h_{r}^{6} \beta_{r}^{6} \left( {I_{r}^{a} + I_{r}^{f} } \right)^{2} }}{{\pi k_{p}^{2} h_{p}^{6} \beta_{p}^{4} \left( {I_{p}^{a} + I_{p}^{f} } \right)^{2} }}} \right]L.$$

T﻿his expression for *z*_*transition*_ based on entropy considerations contrasts with the approach of Bejan, et al.^1^, (copied from Sfeir)^8^ who simply equate the two velocity expressions (Eqs. 9 and 20) evaluated at the plane or axis of symmetry (*f*_*p*_ = *f*_*r*_ = 1) to find

31 $$z_{transition} = \left( {\frac{{4h_{r}^{2} }}{{\pi h_{p}^{2} }}} \right)L.$$

with equal starting momentum. Both expressions show the transition point to be linearly proportional to the slot length of the rectangular nozzle as quoted by Bejan, et al., as the evidence of their approach for turbulent jets^1^. However, the coefficient in Eq. (30) evaluates to 32.8–64.3, showing that entropy would predict a later transition than the 9.6 suggested by Bejan, et al., or 8.62–9.57 using the values cited here (see Fig. 2).

Bejan, et al., argue that the constructal view is about “configurations that offer greater flow access over time”^1^. In this sense, their selection of the free jets is curious. The free jet is termed free because of the absence of significant flow resistance, removing this argument from tuning flow patterns. Because the free jet conserves momentum, flow access may be described in terms of the jet spreading. Balancing the lateral spread of a planar jet, 3*β*_*p*_*zL* (neglecting spreading in *y*), versus the radial spread of a round jet, π(3*β*_*r*_*z*)^2^/4, suggests a transition from planar to circular jets but at a location different than that proposed by Bejan, et al.^1^ at

32 $$z_{transition} = \left( {\frac{{4\beta_{p} }}{{3\pi \beta_{r}^{2} }}} \right)L .$$

T﻿his coefficient takes on values of 5.1–6.1 using values cited here.

Experimentally, Deo et al.^7^ and Sfeir^8^ typically do not identify a simple transition between jet symmetries but find departure from Eq. (9) at meaningfully smaller values than adoption of Eq. (20), between which neither power law holds. Deo et al.^9^ find these two transitions at *z*_*transition*_/*L* = 1.1 and 2.2, respectively. Re-analysis of the results of Sfeir^8^ finds the first transition at *z*_*transition*_/*L* = 1.8–3.4 and the second transition at *z*_*transition*_/*L* = 5.9–8.0. When the two transitions coincide, they occur at *z*_*transition*_/*L* = 7.9–9.5. In either case, entropy considerations appear to not do a better job of predicting the transition between jet symmetries than the simple velocity matching of Sfeir and Bejan, et al.

The analysis above assumed the jets to be self-similar. Sangras and Faeth^10^ show that the uncertainty associated with use of the self-similar assumption is approximately less than 30% at 10 nozzle diameters for a round turbulent nonbuoyant jet and negligible by 30 nozzle diameters. Quinn’s experiments with slot jets show deviation from similar streamwise velocity profiles only at or below 5 equivalent diameters^11^. For conditions in Fig. 2, the transitions occur at > 30 equivalent nozzle diameters. Even with modest uncertainty (note the log scale in Fig. 2a), the conclusion that entropy considerations do not predict jet symmetry transitions does not change.

## Conclusion

In summary, determining when a jet flow transitions from one symmetry to the next is an important but incompletely resolved problem. We find that entropy considerations presented do not predict the transition from planar to round jet. Therefore, the assertions of Sfeir^8^ remain in force. Velocity matching “should be used only as a rough indication of the location of transition”, and the “mechanism of transition” from planar to cylindrical symmetry “is not due to a simple redistribution of momentum”^8^. More advanced approaches that evaluate the stability of jet structures may be helpful, including nonlinear, weakly nonlinear, or Lyapunov-type stability approaches.

## Data Availability

All data analyzed is publicly available as cited in the text.
